# Advances and potential of regenerative medicine in pediatric nephrology

**DOI:** 10.1007/s00467-023-06039-0

**Published:** 2023-07-03

**Authors:** Gisela G. Slaats, Junyu Chen, Elena Levtchenko, Marianne C. Verhaar, Fanny Oliveira Arcolino

**Affiliations:** 1 Department of Nephrology and Hypertension, Regenerative Medicine Center Utrecht, University Medical Center Utrecht, Utrecht, The Netherlands; 2 Department of Development and Regeneration, Cluster Woman and Child, Laboratory of Pediatric Nephrology, KU Leuven, Leuven, Belgium; 3 Department of Pediatric Nephrology, Emma Children’s Hospital, Amsterdam University Medical Centers, Amsterdam, The Netherlands; 4 Emma Center for Personalized Medicine, Amsterdam University Medical Centers, 1105 AZ Amsterdam, The Netherlands

**Keywords:** Pediatric nephrology, Kidney, Regenerative medicine

## Abstract

The endogenous capacity of the kidney to repair is limited, and generation of new nephrons after injury for adequate function recovery remains a need. Discovery of factors that promote the endogenous regenerative capacity of the injured kidney or generation of transplantable kidney tissue represent promising therapeutic strategies. While several encouraging results are obtained after administration of stem or progenitor cells, stem cell secretome, or extracellular vesicles in experimental kidney injury models, very little data exist in the clinical setting to make conclusions about their efficacy. In this review, we provide an overview of the cutting-edge knowledge on kidney regeneration, including pre-clinical methodologies used to elucidate regenerative pathways and describe the perspectives of regenerative medicine for kidney patients.

## Introduction

Chronic kidney disease (CKD) in children poses a high burden for patients and their families and is a global health care problem. CKD is defined as abnormal kidney function that is present for more than 3 months with implications to health [1]. The childhood incidence of CKD in Europe is estimated to be around 11–12 per million of age-related population (pmarp) for stages 3–5, and its prevalence is around 55–60 pmarp [2, 3]. Both the incidence and the prevalence of CKD are higher in males due to the high frequency of congenital abnormalities of the kidney and urinary tract (CAKUT) [2]. Incomplete recovery from acute kidney injury (AKI) can also result in CKD; however, developmental defects and hereditary diseases are the main causes of CKD from birth until the age of 4. Between 5 and 14 years, hereditary diseases, nephrotic syndrome, and systemic diseases most frequently underlie permanent kidney damage. From 15 until 19 years, mainly glomerular diseases are responsible for the onset of CKD in adolescents and young adults [3]. CKD is a progressive disease that cannot be effectively treated. It is associated with numerous comorbidities, impact on child development and decreased quality of life, especially in those with kidney failure in need of kidney replacement therapy (KRT) [4]. The median incidence of KRT in children (0–19 years) is around 9 pmarp, and the prevalence is around 65 pmarp worldwide [2]. Kidney transplantation is currently the best solution for patients with kidney failure, but it is associated with a shortage of donor organs and therefore long waiting lists, risk of rejection, and limited lifetime of the donor organ. From the clinical perspective, there is a high need for novel treatments for pediatric kidney patients.

Regenerative medicine has emerged as an important research field during the past decade focusing on disease modeling and improving, renewing, or replacing tissue function. While genetic kidney diseases are a prevalent cause of CKD in children, genetically modified mouse models do not fully replicate human physiology; therefore, stem cell–derived systems emerge as a promising tool for studying human disease mechanisms and drug testing [5]. Additionally, various strategies have already been used to boost the endogenous regenerative capacity of the kidneys, or to create replacement of organs using organoids (Table 1) and 3D bioprinting. However, the kidney is an extremely challenging organ because of its anatomic and cellular complexity. Therefore, before being able to regenerate the kidney, a detailed understanding of kidney development and kidney repair mechanisms is essential as these processes seem to be interconnected.

**Table 1:** Definitions widely used in regenerative medicine

Regeneration	Generation of new nephrons to replace/renew damaged nephrons leading to effective functionality
Repair	Restoration of damaged cells or nephrons leading to effective functionality
iPSC	Induced pluripotent stem cells are somatic cells that are reprogrammed by overexpression of 4 key transcription factors (*cMyc*, *Sox2*, *Klf4*, *Oct4*) into pluripotent stem cells
ASC	An adult stem cell (or tissue stem cell) remains undifferentiated and can replace cells after cell division (multipotent). Their self-renewal capacity is a key characteristic
MSC	A mesenchymal stromal cell is an adult stem cell that can differentiate into other cell types (multipotent). Human sources of MSCs, which are of stromal origin, include bone marrow, umbilical cord tissue, adipose tissue, and amniotic fluid
KSPC	Kidney stem/progenitor cells represent a unique population of stem cells derived from developing kidneys. They self-renew, express markers of nephron progenitors, and can differentiate into mature epithelial cells of the kidney. They can be derived from fetal kidneys or urine of neonates born prematurely
Organoids	Organoids are self-organized 3D tissue cultures of stem cells and have self-renewal and differentiation capacity. They contain multiple organ-specific cell types which spatially organize and can recapitulate organ function
Tubuloids	Tubuloids are ASC-derived organoids consisting of polarized proximal tubule, loop of Henle, distal tubule and collecting duct cells, representing the kidney tubular epithelium
Secretome	The secretome is the total set of molecules secreted by cells and consists of bioactive molecules such as cytokines/chemokines and growth factors
EV	Extracellular vesicles are secreted by cells with a variable cargo composition. They originate from the endosome or plasma membrane and can establish cell–cell communication

## How kidney development connects to the mechanism of regeneration

Knowing the complexity of the mature kidney, one can marvel at its humble beginning. The current knowledge of kidney development is based on decades of studies in animal models such as zebrafish, frogs, mice, and rats. In this review, we will focus on human kidney development and its relation with regeneration.

**Fig. 1:**
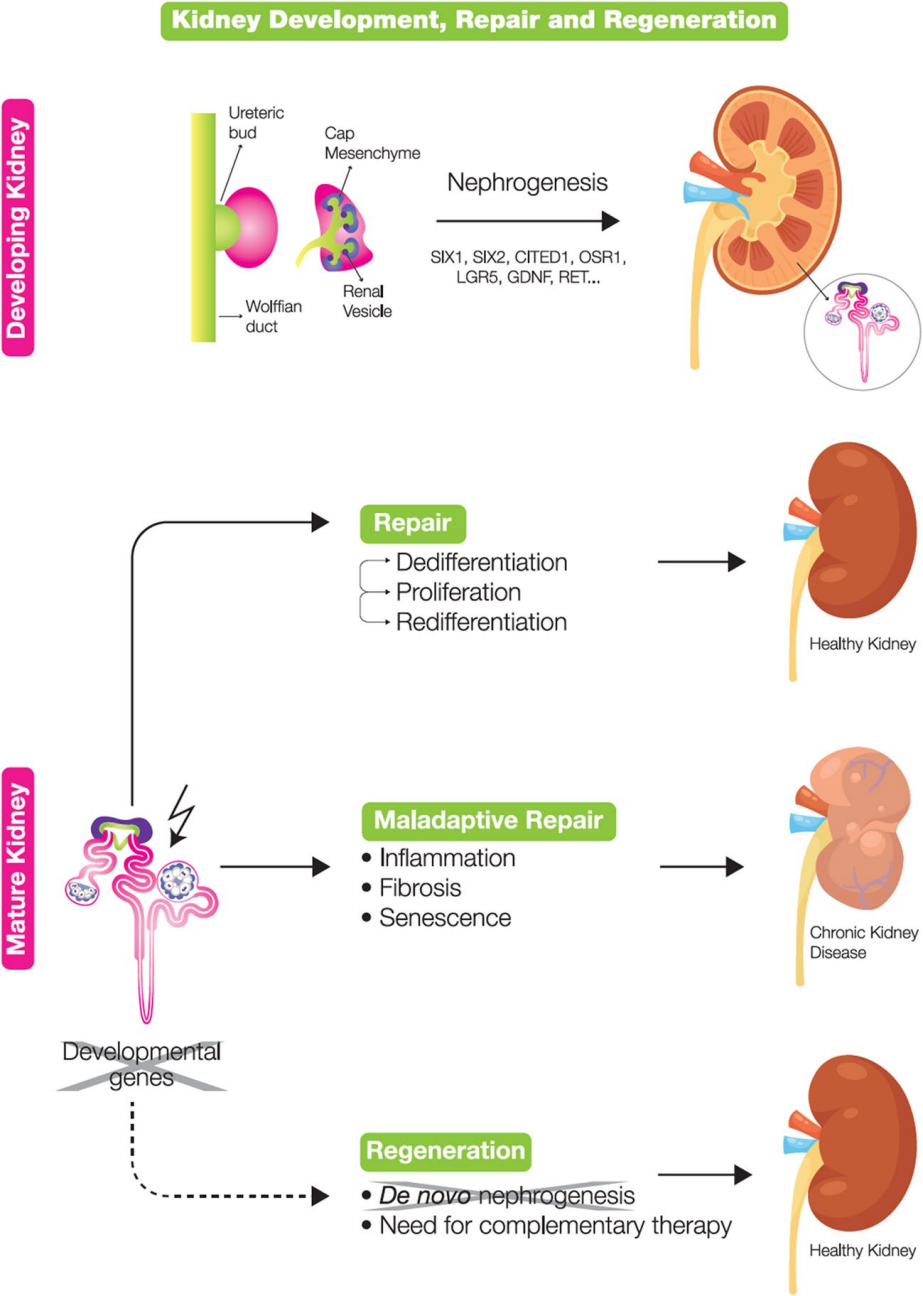
Kidney development, repair, and regeneration. Development: In kidney development, growth and branching of ureteric bud (UB) is influenced by the kidney stem/progenitor cell population localized at the cap mesenchyme (CM). With branching of UB, renal vesicles (RV) emerge by pre-tubular aggregates. Specific genes are upregulated only during nephrogenesis including *SIX1*, *SIX2*, *CITED1*, *OSR1*, *LGR5*, *GDNF*, and *RET*. Repair: After AKI, epithelial cells will dedifferentiate, proliferate, and redifferentiate to form new tubular cells. If the epithelial cells fail to fully re-differentiate, maladaptive repair will occur. Maladaptive repair will cause fibrosis, inflammation, and senescence which will eventually lead to CKD. Regeneration: Genes related to human development including *PAX2*, *LHX1*, and *SOX9* are re-expressed in injured tubular cells during regeneration. However, specific nephrogenesis genes are not upregulated in regeneration, and therefore, complementary therapy is required

The human kidney originates from a succession of three stages: pronephros, mesonephros, and metanephros. The latter is the final kidney prototype, and it arises through cell-to-cell interactions and signaling pathways between the metanephric mesenchyme (MM) and the ureteric bud (UB), both deriving from the intermediate mesoderm [6]. The interplay of both structures is greatly influenced by the production of nephrogenic factors derived from a restricted kidney stem/progenitor cell (KSPC) population (Table 1) localized at the cap mesenchyme (CM) (Fig. 1). Expression of the transcription factor *SIX2* (Sine Oculus Homeobox Homolog 2) is a determinant feature of KSPC, since *SIX2* + KSPCs will give rise to all cell types of the nephron [7].

Branching of the UB tree is accompanied by the release of several factors such as *Wnt9b* and later *Wnt4*, and the concomitant downregulation of *SIX2* gradually transits KSPCs into epithelial cells through a process of mesenchymal-to-epithelial transition (MET). Consequently, these epithelial cells form the pre-tubular aggregates from which the renal vesicles (RVs) emerge [6]. The RV evolves into a comma-shaped body and S-shaped body. Endothelial cells migrate into the cleft of the S-shaped body to assist in the formation of the renal corpuscle. The upper part of S-shaped bodies fuses with what has been the UB, to enable the connection to the collecting duct [8]. As a result of epithelial differentiation, the glomerular capsule, the podocytes, the descending and ascending limb, and the distal tubules are formed, all originating from the *SIX2* + KSPC. Next, the kidney vasculature emerges in close contact with other structures of the nephron. When ready, the podocytes encapsulate the glomerular capillaries, and the two structures fuse to the glomerular basement membrane. The cells surrounding the nephrons (interstitial cells) originate from another pool of progenitor cells, which are *FOXD1* + (Forkhead Box 1), localized at the top of the CM [9]. Stemming from the UB, larger kidney structures emerge: the collecting system and the renal pelvis. In humans, by the 36th week of gestation, the kidneys are functionally mature, and the *SIX2* + KSPC population is exhausted; therefore, no new nephrons are formed [10], which limits the regenerative potential of the organ (Fig. 1). Children born premature are at increased risk of CKD, likely due to decreased nephron number and exposure to post-natal nephrotoxins. They are also at higher risk of neonatal acute kidney injury (nAKI), which may further decrease the viable nephron number and potentiate progression to CKD [11].

## Kidney regeneration after birth

What do we know about the behavior of kidney cells after injury and how does this relate to potential therapeutic approaches?

Upon AKI, proximal tubular cells rapidly lose their brush border and dedifferentiate into a mesenchymal phenotype. Processes like cell migration, detachment, apoptosis, and necrosis result in denudation of the tubular basement membrane. In the glomerulus, injury can lead to podocyte loss with a complete glomerular collapse when the podocyte cell number falls below 20% of the original amount [12]. The capacity of the kidney to functionally regenerate upon AKI is a major determinant of the outcome, but no specific therapeutic approach has been shown to improve the effectiveness of regeneration so far [13]. Following AKI, a variety of intrinsic repair processes are activated rapidly, but repetitive or prolonged injury may lead to an unwanted maladaptive regenerative process. Maladaptive repair of tubular cells occurs when epithelial cells fail to fully re-differentiate or become growth arrested in G2 cell cycle phase, becoming an additional source of profibrotic factors, inflammation, and senescence leading to CKD and eventual kidney loss [14] (Fig. 1). Mechanisms of (limited) kidney regeneration have been an ongoing debate in the stem cell research field during the last decades. Whereas many studies attempted to identify cells with the ability to generate nephrons, growing evidence shows that the regeneration process arises through phenotypic and metabolic plasticity of tubular cells mediated by the microenvironment. Previously, it has been postulated that bone marrow-derived stem cells could translocate into the kidney upon damage, but that has not been substantiated, and the possibility of regeneration driven by extrarenal cells has been disregarded [15, 16].

Although it has previously been suggested that a specific population of CD133 + CD24 + , Vimentin + , and PAX2 + cells would function as stem cells in the kidney, displaying self-renewal properties and potential to differentiate into epithelial cells to repair damaged tissue [17, 18], it is now clear that after kidney injury, the repair process is accomplished by remaining reparative cells within the tubule that dedifferentiate, proliferate, and re-differentiate without any contribution from a preexisting specific progenitor cell population [19, 20]. To elucidate this process, cells from mice were genetically labeled during ischemic injury to mark individual damaged tubular cells and to follow subsequent recovery. The number of labeled cells increased significantly upon injury, indicating that tubular epithelium can arise from any surviving tubular cell and not from a fixed progenitor population [21]. The debate goes on to define the exact mechanism of repair and regeneration. Although kidney function is restored to baseline after AKI, patients frequently develop CKD, which demonstrates that re-entering the cell cycle is insufficient to fully regenerate nephrons [22]. This highlights the importance of research and development of techniques to identify target pathways and the factors that drive effective regeneration to boost this process.

## Regeneration versus development

In rodent models, injured tubular cells start to re-express genes and proteins that are active during development, such as *PAX2*, *LHX1*, *SOX9*, followed by increased proliferation rates, release of several growth factors such as epidermal growth factor (EGF), insulin-like growth factor (IGF), and transforming growth factor-β (TGF-β) and involvement of the canonical *Wnt* signaling pathway [23, 24]. A recent study has demonstrated a similar mechanism in humans. Using intercellular cross-talk analysis, it has been shown that upon AKI, tubular epithelial cells activated the transcription factor *SOX9*, and these cells released factors such as VEGF, complement, SPP1, and CALCR, which influenced the surrounding cells to facilitate endogenous repair [25]. In the same study, the authors identified S100 calcium-binding protein 9 (S100A9) as a protein that enhances cell proliferation and might be directly related to tissue regeneration [25].

Still, upregulation of genes that are specific for nephrogenesis, such as *SIX1*, *SIX2*, *CITED1*, *OSR1*, *LGR5*, *GDNF*, and *RET*, has not been reported after injury or during repair (Fig. 1). Therefore, kidney regeneration does not fully recapitulate development.

Recently, it has been shown for the first time that injection of *SIX2* + neonatal kidney stem/progenitor cells (nKSPC) into human deceased donor kidneys induces the de novo expression of *SIX2* in proliferating proximal tubular cells. These cells were derived from the urine of neonates born prematurely; therefore, they endogenously express *SIX2* [26]. nKSPCs were injected via the kidney artery into human grafts that were not used for transplantation and were perfused during 6 h in normothermic machine perfusion (NMP). Besides *SIX2* expression, these kidneys showed upregulation of regenerative markers, such as *SOX9* and *VEGF*, and had significantly lower levels of kidney injury biomarkers and reduced inflammatory cytokines [27]. The reactivation of *SIX2*, a nephrogenic factor, might be related to the initiation of an endogenous regenerative repair of the kidney tissue and can reflect a possibility to therapeutically re-induce nephrogenesis.

Nevertheless, the mechanisms of regeneration are not fully understood, and several technologies have been developed to study and improve the regenerative potential of the kidney tissue for therapeutic purposes.

## Fundamental research pushes forward the field of regenerative medicine

Innovative technologies including genomics, transcriptomics, proteomics, metabolomics — conjunctively referred to as “omics”— have generated large data set collections and analyses to improve our understanding of basic principles and mechanisms of kidney development, repair, and regeneration. Based on knowledge acquired from omics studies, researchers were able to develop the protocols to induce pluripotent stem cells (iPSCs) to form nephrons in vitro, the so-called kidney organoids. Single cell RNA sequencing (scRNA-seq) data was used to optimize the kidney cell differentiation and to reduce the rate of non-kidney cell types [28]. Kidney organoids are nowadays one of the most important in vitro models to elucidate kidney development.

### Transcriptomic characterization of repair and regeneration

Transcriptomic studies are based on RNA sequencing (RNA-seq) technology and indicate transcriptional activity of genes. RNA-seq technology has been used for analyzing kidney biopsies and supports diagnosis and prognosis in some of the kidney diseases. Bulk RNA-seq, scRNA-seq, and single-nuclei RNA-seq (snRNA-seq) are three commonly used RNA-seq techniques. Bulk RNA-seq provides an overview of the average of gene expression. ScRNA-seq and snRNA-seq can provide information about each cell and show the potential to find molecular differences which are only linked to specific cell types. ScRNA-seq can measure both cytoplasmic and nuclear transcripts whereas snRNA-seq can only measure nuclear transcripts. Not many RNA-seq studies have been performed to characterize repair and regeneration in AKI or CKD; however, some studies report molecular characterization of the transition from acute to chronic kidney injury following ischemia/reperfusion injury (IRI). An scRNA-seq study defined key differences in adaptive and fibrotic repair, suggesting potential druggable pathways. The authors found that specific maladaptive/profibrotic proximal tubules (PT) expressed proinflammatory, profibrotic cytokines, and myeloid cell chemotactic factors (e.g. CXCL2, IL1b, CCL3) after long IRI. Additionally, maladaptive PT cells showed a marked enrichment of ferroptosis and pyroptosis. Pharmacological targeting of pyroptosis/ferroptosis (VX-765, pyroptosis inhibitor and liproxstatin, ferroptosis inhibitor) in vivo induced cells towards adaptive repair and improvement of fibrosis [29]. This supports the potential of RNA-seq for the identification of regenerative therapeutic targets [30]. SnRNA-seq analysis of biopsies of 8 individuals with severe AKI revealed common epithelial cell response patterns including oxidative stress, hypoxia, interferon response, and epithelial-to-mesenchymal transition [31]. Similarly, scRNA-seq of urine samples demonstrated that urinary cells in adaptive states are potentially derived from the thick ascending limb and show regenerative signatures by expressing *PAX2*, *SOX4*, and *SOX9*, which were predominantly expressed in the presumed progenitor clusters [32]. These outcomes underline the possibility of applying RNA-seq technology in humans, but still leave a gap between research and clinical application.

### Metabolomic characterization of repair and regeneration

Besides transcriptomics, metabolomic studies may reveal the molecular signatures of cells and tissues upon injury and repair. A very promising advance in metabolic pathways has been made by using isotope tracing in dynamic metabolic processes [33] based on matrix-assisted laser desorption/ionization mass spectrometry imaging (MALDI-MSI). This technology might provide a truly comprehensive understanding of the interplay between biochemical alterations and cell type-specific functions, metabolic fluxes, and dynamic interpretations of cellular states [34]. Using this technology in a mouse model of AKI, authors concluded that the maladaptive repair in tubular cells is characterized by differences in production of lactate, which could possibly be the result of higher glycolytic activity and, as injury progresses, a concomitant reduction of the tricarboxylic acid (TCA) cycle metabolite consumption [34]. Over the past decades, metabolomics has added a promising number of new biomarkers through better pathophysiology knowledge, paving the way for insightful perspectives on the management of different kidney diseases [35]. However, the metabolome greatly varies with age, diet, drug consumption, lifestyle, and, in adults, with gender, making it difficult to compare studies in adults and in children and neonates. More metabolomic studies focused on pediatric patients are required to determine the practical clinical impact of metabolomics in conditions of kidney damage and repair.

## Kidney organoids and their potential for clinical implementation

In the field of regenerative medicine, organoid cultures are widely used to model disease, study physiology, and develop clinical applications, like drug screening and personalized medicine approaches, as well as their use for regenerative therapies. Organoids are self-organized 3-dimensional (3D) tissue cultures of stem cells, which have self-renewal and differentiation capacity. They contain multiple organ-specific cell types which spatially organize and can recapitulate organ function (Table 1). Here, we will describe two types of kidney organoid models which have been developed in the past 5 years: iPSC-derived kidney organoids and adult stem cell (ASC)-derived tubuloids (Table 1).

### iPSC-derived organoids

iPSC-derived organoids have emerged as advanced in vitro models of kidney development, physiology, and disease. iPSC-derived organoids reflect mainly kidney developmental aspects by mimicking nephrogenesis. When iPSCs are differentiated into kidney organoids, most of the nephron segments are present: both proximal and distal tubule cells, glomerular structures, and loop of Henle as well as collecting duct cells can be found [36]. Recently, novel hybrid protocols have been designed to culture podocyte-like cells in kidney iPSC-derived organoids [37]. Patient mutations can be created by genome-editing technologies like CRISPR/Cas9 in order to model diseases, or patient-derived cells can be reprogrammed to have pluripotent features [38] (Table 2). To date, iPSC-based disease modeling has successfully been used for studying genetic kidney diseases such as cystinosis [39], nephronophthisis (NPH), and nephrotic syndrome [27, 40, 41], as well as for drug development (Table 2). Nevertheless, there are several limitations of using kidney iPSC-derived organoid models. The organoids most closely resemble human embryonic kidney tissue and not a mature organ [36]. In addition, a vascular system is missing [36]. So far, both endothelial cells (CD31 +) and interstitium could be induced in kidney organoid culture [42]; however, this still needs to be further developed into more mature and functional vascularization. Recently, iPSC-derived vascular organoids showed to be a new cell source of functional and flow-adaptive vascular cells for the creation of a perfused macrovessel model [43], which might inspire future vascularized kidney organoid culture. This new model recapitulates the bi-layer vessel architecture and allows in vitro studies of vascular disease [43]. Unfortunately, after directed differentiation protocols toward kidney organoids, still 10–20% off-target non-renal cells appear in culture, including mainly neuronal-like and muscle-like cell populations. Protocols on how to improve iPSC-derived organoid cultures are under development [44]. For example, iPSC organoid-derived tubuloid cultures showed disappearance of immature and off-target cell populations, accessibility of the apical site, and prolonged expansion capacity [45]. Notably, iPSC-derived organoids generated by knocking out a single gene of interest miss potential genetic or epigenetic modifiers that can be present in individual patients. On the other hand, this provides a model to identify potential modifiers when comparing patient-derived organoids with gene-edited iPSC-derived organoids.

**Table 2:** iPSC-derived organoid or tubuloid models in pediatric genetic kidney diseases

Disease	Gene	Approaches	Applications	Reference
ARPKD	*PKHD1*	Patient iPSC-derived kidney organoids	Drug validation in a patient-specific manner	[78]
ARPKD	*PKHD1*	iPSC-derived organoid-on-a-chip model subjected to flow	Therapeutic target discovery using transcriptomics	[79]
ARPKD	*PKHD1*	iPSCs harboring loss-of-function mutations that recapitulate cystic phenotype	Disease modeling	[80]
NPH-RC	*IFT140*	Patient-derived iPSC kidney organoids, gene corrected	Disease modeling	[81]
Cystinosis	*CTNS*	Patient urine-derived tubuloid model	Personalized drug screening	[47]
Cystinosis	*CTNS*	iPSC-derived organoids	Drug testing	[39]
CAKUT	*WT1*, *HNF1β*, *GLI3*, *COL4A3*	Urine-derived patient-specific iPSCs and kidney organoids	Disease modeling	[82]
Nephrotic syndrome	*NOS1AP*	Human kidney organoids with knock-in of patient specific variant	Disease modeling	[83]
Nephrotic syndrome	*NPHS1*	Organoid-derived glomeruli and podocytes	Disease modeling	[84]
Nephrotic syndrome	*NPHS1*	Patient-derived iPSC organoids	Disease modeling	[40, 41]

*ARPKD* autosomal recessive polycystic kidney disease, *NPH-RC* nephronophthisis-related ciliopathy; *CAKUT* congenital anomalies of the kidney and urinary tract

### Tubuloids

**Fig. 2:**
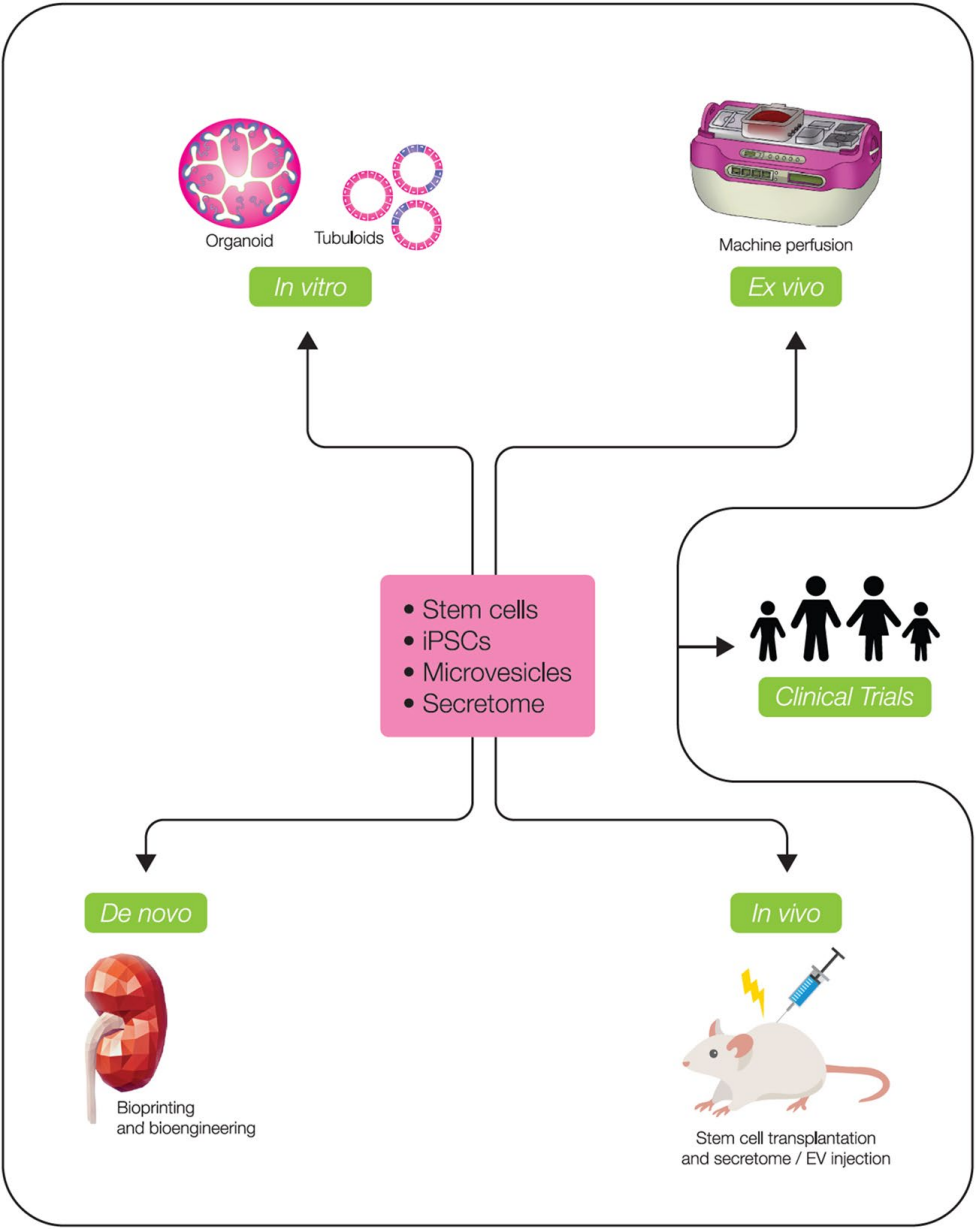
State-of-the-art tools applied in regenerative medicine approaches. In vitro: Kidney organoid cultures generated from iPSC can be used for modeling development, and together with tubuloids are valuable tools to model diseases and to test drugs. Additionally, iPSC-derived kidney organoids have potential to replace damaged kidney tissue. Ex vivo*:* Machine perfusion can serve as a suitable platform to locally boost the regenerative potential of donor kidneys having inferior quality prior to transplantation by means of cell therapy or drug injection. De novo*:* Bioprinting platforms can facilitate high throughput culture of highly consistent and reproducible organoid cultures to build kidney tissue. In vivo*:* Therapy including stem cell transplantations, cell secretome injection, and extracellular vesicles (EVs) injection can be used clinically to induce repair and regeneration, as already shown in pre-clinical models. All these methodologies are fundamental to elucidate the processes of kidney repair and regeneration prior to clinical trials and clinical applications

Kidney organoids can also be generated from ASCs obtained either from kidney biopsy material or from urine, and thus carry the exact genetic and epigenetic information of the patient. These 3D structures are called tubuloids (Table 1) as they mainly represent the tubular epithelium and lack differentiation into glomerular cells. In tubuloid cultures, podocytes and stroma are absent, and like in iPSC-derived organoids, the vasculature is also absent in tubuloids. Tubuloids can be long-term expanded, without the need of genetic modification and without the risk of off-target differentiation [46]. To date, genome-editing protocols in tubuloids have not yet been published. Recently, tubuloids derived from urine of cystinosis patients were used to develop novel treatment strategies [47] (Table 2), indicating their power as a disease model for translational applications. In this study, an omics-inspired drug screen revealed a novel combination therapy, which has been tested in patient-derived tubuloids. Age- and gender-matched healthy donor-derived tubuloid cultures are used as controls. Biobanks of healthy donor and patient material-derived tubuloids will facilitate the development of personalized medicine and will create a short line from bench to bedside. Kidney organoids can be cultured non-invasively from urine from (pediatric) kidney disease patients, as being long-term expandable and genetically stable cultures. For pediatric nephrology, a urine cell-derived tubuloid biobank [48] will be of interest to study hereditary kidney disease. For children and adolescents, non-invasive ways of collecting primary patients’ cells are preferred. As an advancement of primary urine cell cultures in 2D [49], the 3D tubuloid cultures will provide an improved cell culture model for fundamental and translational research, including drug development (Fig. 2). For example, drug screening on tumor organoids derived from childhood malignancies showed successful identification of potential therapeutic agents targeting pediatric tumors [50]. Kidney organoid cells can also be cultured in flow chambers [51]. The introduction of flow in perfused cell systems of tubuloid cells on a chip reflects another way to create a more advanced in vitro kidney model for research applications [51]. ASC-derived organoids were first developed to model murine intestine [52]. To date, one key application of the intestinal organoids is the development of forskolin swelling assays allowing drug response monitoring in cystic fibrosis patients. In vitro swell responses can be monitored and correlate with the individual’s clinical response to therapy [53]. This is an important showcase stressing the value of organoids for clinical applications.

### Application of organoids in future transplantable kidney tissue

Kidney organoid cultures are also further investigated in the direction of replacing damaged kidney tissue (Fig. 2). Several attempts have been made to create functional kidney structures by transplanting iPSC-derived organoids in mice. Organoid transplantation under the kidney capsule or subcutaneously resulted in functional glomerular perfusion, connection to vascular networks or vascularization, and improved morphogenesis [54, 55]. In addition, iPSC-derived nephron sheets which contain many nephrons could be transplanted in immunodeficient mice, and this scalable protocol was demonstrated to produce kidney tissue with glomerular filtration function [56]. A critical step in future experiments is to establish a robust connection between the transplanted organoids and the host’s vasculature, and to overcome graft overgrowth by stromal cells in the long term [57]. To further optimize kidney organoid engraftment, material-driven applications refine organoid culture by implementing improved hydrogel engineering [58]. Soft hydrogels demonstrate better performance of kidney organoids in comparison to stiff hydrogels [58, 59]. 3D bioprinting of kidney matrix could facilitate high throughput culture of highly consistent and reproducible organoid cultures in transplantation-compatible hydrogels in the future. Despite gaining novel fundamental knowledge, no clinical applications in pediatric nephrology of kidney organoids or tubuloids have been reported so far. Nevertheless, over the long term, kidney organoids might have the potential to advance KRT and, as an outlook, have a significant contribution to the bioengineering of kidney grafts and of bioartificial kidney tissue. Implantable bioartificial kidneys, which are attached to the systemic circulation, are not yet clinically tested, as both technical and biological challenges must be overcome. The development of a transplantable auxiliary kidney would hold great promise for people with kidney disease by (partly) implementing kidney function. In comparison to wearable and portable dialysis machines, which are cell-free, a bioartificial kidney would overcome dialysis shortcomings characterized by poor clinical outcomes and low quality of life. Functional requirements, which need to be addressed, include membrane characteristics, cell characteristics, and functional aspects like toxin excretion and nutrient and water reabsorption capacity. Finding cellular components that are capable of taking over tubular functions in a bioartificial kidney and which are biocompatible at the same time will substantially advance its development [60].

## The current clinical applications of regenerative medicine: the status of stem cell transplantation and extracellular vesicle injection

In preclinical models of kidney injury, repair and regeneration have been observed upon different types of (stem) cell transplantations, cell secretome injection, and extracellular vesicle (EV) injection (Fig. 2). However, translation of these methodologies to the clinics still faces several challenges.

### Cell therapy-based clinical trials

Mesenchymal stromal cells (MSC) (Table 1) have immunomodulatory properties and might play a role in tissue repair, therefore being the most widely used type of cells for cell therapy of damaged kidneys. A few clinical trials have been performed showing feasibility and safety of MSC infusion in patients [61–64]. In an 18-month follow-up study, seven patients with CKD of different etiologies such as hypertension, nephrotic syndrome, and unknown etiology were infused with 1–2 million autologous bone marrow MSC/kg for safety and tolerability evaluation. Although none of the patients had adverse events related to the therapy, kidney function (GFR and serum creatinine measurements) did not improve [62]. A similar trial of bone barrow autologous MSC transplantation in 6 ADPKD patients confirmed feasibility of the therapy, but in these patients, serum creatinine levels were significantly improved [63].

In kidney transplantation, MSC therapy shows promising results with potential to induce immunotolerance. In a phase 1 trial, four living-donor kidney transplant patients were given autologous bone marrow MSC 1 day before or 7 days after kidney transplantation while receiving induction therapy. All patients had stable graft function in 5–7-year follow-up, but the protolerogenic effect of MSC was variable. One of these patients was successfully weaned off immunosuppressive drugs and stayed free from anti-rejection therapy with optimal long-term kidney allograft function [65]. In a larger study, patients received MSC infusion 6 and 7 weeks after kidney transplantation in a randomized prospective, single-center, open-label trial. Twenty-nine patients received MSC and had early tacrolimus withdrawal, while 28 patients were in the control tacrolimus group. Early tacrolimus withdrawal with MSC therapy was feasible and safe, and there was no increased rate of rejection. Kidney function was preserved in both groups, but the MSC-treated patients were not prevented from developing progressive fibrosis [64].

These studies suggest that MSC therapy in humans is feasible and safe on a short-term basis, and that the immunomodulatory effect is promising. Nevertheless, MSCs of different tissues are devoid of differentiation capacity into mature kidney epithelial cells, and trials have not shown effective tissue repair or regeneration leading to improved kidney function. Therefore, it remains crucial to find a superior source of (stem) cells to develop effective kidney tissue regeneration, and kidney-derived cells might be the ideal candidate. Autologous selected renal cells (SRC), a pool of proximal tubule and glomerular cells and other cell subpopulations, such as interstitial cells, were used in a trial of 22 patients with advanced type 2 diabetes–related CKD (D-CKD). In this study, the cell therapy seemed to improve kidney function and possibly halted type 2 D-CKD progression [66]. In another safety study, 18 patients with CKD of unknown cause (stages 3–5) were followed for 36 months after receiving a single infusion of angiogenic/anti-fibrotic autologous adipose-derived stromal vascular fraction (SVF) cells into their kidneys bilaterally via renal artery catheterization. Both kidney structure and function were shown to be improved [67].

### *Ex vivo *cell therapy

With the increasing need for kidney transplantation in the adult and pediatric populations, the criteria for organ recruitment have been extended as an attempt to reduce the waiting time by increasing the donor organ pool. This implies that kidneys of older donors (> or = 60 years) or donors who are aged 50 to 59 years and have two of the following three features: hypertension, serum creatinine > 1.5 mg/dl, or death from cerebrovascular accident [68] can be offered to patients. However, these kidneys are of suboptimal quality, rendering grafts more susceptible to ischemia reperfusion injury (IRI) in comparison with organs derived from living healthy donors [69]. These are the targeted organs for receiving ex vivo cell therapy in order to reduce IRI, reduce the immunogenicity of the kidney graft, and promote regeneration of already injured organs.

Ex vivo cell therapy can be performed during machine perfusion (MP), the current method of choice for preserving kidney allografts obtained from deceased donors. The first study of administration of human MSC into human kidneys during MP suggested tissue regeneration by increased cellular proliferative rates and ATP production [70]. Using preterm neonatal KSPC, we have shown that cell therapy of human deceased donor kidneys during normothermic MP (NMP) induced de novo expression of *SIX2* in the donor tubular cells while it also upregulated regenerative genes such as *VEGF* and *SOX9* [26, 27]. This suggested that kidney stem cells or developmental factors released by these cells are necessary to induce endogenous regeneration in kidneys. Still, long-term perfusions will be fundamental to prove effective regeneration of the kidney tissue leading to improved graft function.

### Secretome and EVs

The described beneficial effects of cell therapy have been mainly attributed to paracrine mechanisms [5]. In preclinical studies, the secretome (Table 1) of different kinds of MSCs has shown regulatory function in cell proliferation, cell migration, cell differentiation, and modulation of the immune system. The secretome consists of bioactive molecules such as cytokines/chemokines and growth factors including granulocyte-colony stimulating factor, leukemia-inhibitory factor, macrophage-colony stimulating factor, PGE2, IL-10, TGFβ, IDO, HO-1, HGF, VEGF, FGF, and IGF-1, which can also modulate kidney regenerative responses [70, 71]. MSC-derived secretome was shown to drive kidney regeneration by inducing surviving kidney cells to dedifferentiate and replicate to restore the lost kidney cells in animal models [72, 73].

Being part of the secretome, EVs (Table 1) play an important role in inducing kidney regeneration. EVs are a type of nanoscale vesicles encapsulated by cytomembranes, which have an important function in intercellular communication, and are widely present in the body fluids, including blood, urine, and amniotic fluid. Preclinical research has proven that EVs can improve AKI by inhibiting inflammation, apoptosis, and oxidation and by regulating angiogenesis [73–75].

In human donor kidneys, MSC-EVs were infused during hypothermic oxygenated perfusion (HOPE). HOPE + EV kidneys had lower ischemic damage score and better kidney ultrastructure. They had higher HGF and VEGF levels with lower apoptosis rate than control kidneys. Moreover, HOPE + EV kidneys had lower lactate release and higher glucose levels than controls, suggesting that the gluconeogenesis system was preserved [76].

Only one clinical trial has been performed using EVs in CKD patients. In this study, cell-free cord-blood MSC-derived EVs were administered to 20 CKD patients stage III and IV (eGFR 15–60 mg/ml). The 20 patients in the treatment group exhibited improvement of eGFR, serum creatinine level, and blood urea and urinary albumin creatinine ratio compared with 20 patients in the placebo group at the end of the study period of 1 year [77]. Although the biopsies of some of the treatment group patients did not show significant histologic changes, the expression of Ki67 (a marker of proliferation) in some tubular cells confirmed the ability of MSC-EVs to activate tubular cells [77]. Longer observation and larger number of patients are required to demonstrate the efficacy and safety over the long run of EV injection as a treatment inducing kidney regeneration.

## Future perspectives

Regenerative medicine is a rapidly evolving field. Here, we describe the current state of knowledge and understanding of kidney development, repair, and regeneration. Furthermore, we provide an overview of methodologies to understand and potentially enhance kidney regeneration. Unfortunately, clinical readiness of applications based on preclinical findings using omics, stem cells, or bioartificial transplants has not yet been achieved. However, the field is rapidly moving forward, and detailed knowledge about kidney development, repair, and regeneration paves the way for novel translational solutions for kidney patients (Fig. 2). Nephron-like structures are being cultured in 3D and hold promise for advanced modeling of kidney (patho)physiology, drug screening, and personalized medicine, as well as for regenerative therapies as part of a bioartificial kidney or transplantable kidney tissue. Available omics technologies are propelling our understanding of the mechanisms of kidney injury and repair, which opens opportunities for finding new druggable targets or interventional gene/cellular therapies to finally improve the outcome of kidney disease.

Currently, there are no FDA-approved stem cell-based therapies for CKD. Nevertheless, clinical trials to test efficacy and safety of stem cell-based therapies for kidney disease are being conducted. In the future, it will be important to also conduct clinical trials specifically designed for children to improve outcomes and advance knowledge. There is no doubt that clinical trials including children aiming to ameliorate CKD or find a cure for kidney failure are facing many insecurities. In addition, ethical aspects of these novel regenerative medicine therapies have to be carefully considered. Regarding the long-term goals to create donor organs and to develop personalized regenerative medicine approaches, there is a long way to go. Nevertheless, every day, we get closer to regenerative solutions for kidney patients.

## Key summary points

1. During kidney development, functional nephrons are formed; however, this process cannot be repeated during repair and regeneration in a mature kidney.
2. Advanced basic science approaches and advanced organoid cell culture models enable understanding mechanisms of kidney tissue repair and regeneration.
3. Cell(-based) therapy is currently being tested for regenerative medicine applications. However, these cell therapies are not yet available in the clinic for kidney failure patients.
4. Generation of a bioartificial kidney is on the horizon; however, more knowledge about kidney development, repair, and regeneration is required for future progress.
